# Extracellular Vesicles Secreted by Corneal Myofibroblasts Promote Corneal Epithelial Cell Migration

**DOI:** 10.3390/ijms23063136

**Published:** 2022-03-15

**Authors:** Vincent Yeung, Tancy C. Zhang, Ling Yuan, Mohit Parekh, John A. Cortinas, Eleni Delavogia, Audrey E. K. Hutcheon, Xiaoqing Guo, Joseph B. Ciolino

**Affiliations:** 1 Department of Ophthalmology, Schepens Eye Research Institute of Mass Eye and Ear, Harvard Medical School, Boston, MA 02114, USA; 20080017@nxmu.edu.cn (L.Y.); mnparekh@meei.harvard.edu (M.P.); audrey_hutcheon@meei.harvard.edu (A.E.K.H.); xiaoqing_guo@meei.harvard.edu (X.G.); joseph_ciolino@meei.harvard.edu (J.B.C.); 2 Independent Researcher, Boston, MA 02115, USA; tczhang@bu.edu; 3 Division of Newborn Medicine & Department of Pediatrics, Boston Children’s Hospital, Harvard Medical School, Boston, MA 02115, USA; john.cortinas@childrens.harvard.edu (J.A.C.); eleni.delavogia@childrens.harvard.edu (E.D.)

**Keywords:** cell–cell communication, cornea, extracellular vesicles (EVs), epithelial cells, fibroblast, keratocytes, myofibroblast, migration, proteomics, wound healing

## Abstract

Corneal epithelial wound healing is a multifaceted process that encompasses cell proliferation, migration, and communication from the corneal stroma. Upon corneal injury, bidirectional crosstalk between the epithelium and stroma via extracellular vesicles (EVs) has been reported. However, the mechanisms by which the EVs from human corneal keratocytes (HCKs), fibroblasts (HCFs), and/or myofibroblasts (HCMs) exert their effects on the corneal epithelium remain unclear. In this study, HCK-, HCF-, and HCM-EVs were isolated and characterized, and human corneal epithelial (HCE) cell migration was assessed in a scratch assay following PKH26-labeled HCK-, HCF-, or HCM-EV treatment. HCE cells proliferative and apoptotic activity following EV treatment was assessed. HCF-/HCM-EVs were enriched for CD63, CD81, ITGAV, and THBS1 compared to HCK-EV. All EVs were negative for GM130 and showed minimal differences in biophysical properties. At the proteomic level, we showed HCM-EV with a log >two-fold change in CXCL6, CXCL12, MMP1, and MMP2 expression compared to HCK-/HCF-EVs; these proteins are associated with cellular movement pathways. Upon HCM-EV treatment, HCE cell migration, velocity, and proliferation were significantly increased compared to HCK-/HCF-EVs. This study concludes that the HCM-EV protein cargo influences HCE cell migration and proliferation, and understanding these elements may provide a novel therapeutic avenue for corneal wound healing.

## 1. Introduction

The cornea is the most anterior tissue of the eye that transmits light and provides protection to the intraocular eye components [1]. The outermost layer consists of the corneal epithelium, which constitutes the main barrier of protection against injury and infection by foreign pathogens, fluid loss, and physical and chemical trauma [2]. During corneal epithelial injury, corneal healing occurs in a multifaceted approach to avoid infection [3]; however, defects in the ability to repair and restore epithelial integrity post-injury can damage the underlying corneal stroma, leading to diminished corneal transparency and vision loss [4]. 

The corneal stroma, which is the backbone of the cornea, is composed of collagenous lamellae consisting of tightly packed collagen fibrils embedded in a matrix of glycoproteins (GPs) and proteoglycans (PGs) [5]. The parallel arrangement and uniform spacing that runs orthogonally are thought to give strength and promote corneal transparency [3,6]. The (human) corneal keratocytes (HCKs) are a cell population of neural crest-derived, quiescent mesenchymal cells of the stroma [7], responsible for the secretion of the stromal extracellular matrix (ECM), including collagen fibrils and PGs. Upon stromal injury, HCKs are capable of transforming into a repair-phenotype of activated human corneal fibroblasts (HCFs) [6]. HCFs are a proliferative and migratory cell type that produce repair-type ECM components, including fibronectin (FN1), proteinases, and cell–ECM adhesion molecules (integrins) [8,9]. Moreover, some HCFs can further differentiate to human corneal myofibroblasts (HCMs) under the synergistic action of serum and transforming growth factor β1 (TGF-β1). HCMs are characterized by increased cell size and alpha smooth muscle actin (αSMA) expression [10,11,12]. HCMs are responsible for the increased secretion of matrix metalloproteinases (MMPs), collagenases, and chemokines that govern ECM remodeling and collectively mediate wound closure [13]. Nevertheless, persistent HCM onset can lead to corneal scarring and the loss of corneal transparency. 

In the cornea, bidirectional communication between the corneal epithelium and stroma plays a critical role in corneal wound repair [14], and it is increasingly understood that cell–cell communication is vital for this process. Precisely, extracellular vesicles (EVs), which are paracrine mediators, have become an area of growing interest in corneal tissue biology in relation to physiological and pathological responses to wound healing. Early research from our laboratory provided initial evidence that EVs are released into the corneal stroma following a keratectomy [15]. Our investigations have found that epithelial EVs released by the corneal epithelium can promote neovascularization and are involved in corneal wound healing [16]. More recently, we have shown that human corneal epithelial (HCE) cell-derived EVs encapsulated provisional matrix proteins that can trigger fibroblast differentiation into myofibroblasts [11]. These studies revealed different roles for HCE-EVs on the corneal stroma; however, there remains a knowledge gap on how HCK-, HCF-, and HCM-EVs (termed corneal stromal EVs in this study) affect the corneal epithelium during corneal wound healing.

To date, our understanding of stromal cells, such as adipocytes, fibrocytes, fibroblasts, and mesenchymal stem cells (MSCs), along with their secreted EVs, has been reported in studies that mediate disease-exacerbating changes in asthma [17], cancer [18,19,20,21,22], fibrosis [23,24,25], and cardiovascular diseases [26,27,28], but also show the therapeutic action of MSC-EVs by targeting different tissues, including the lungs, placenta, and thymus [29,30,31,32,33,34,35,36,37]. By focusing on the cornea, studies have investigated how corneal MSC-EVs can improve corneal epithelial wound healing, reduce corneal epithelial defects, and promote scarless stromal recovery post-corneal injury in vivo [38,39,40,41]. These studies revealed a novel application of EVs as therapeutic carriers of cargo that promotes corneal tissue regeneration. These data support the idea that HCK-, HCF-, and HCM-EVs remain complex and their mechanistic influence on corneal wound healing in the corneal epithelium remains unknown. 

In this study, we isolated EVs from HCKs, HCFs, and HCMs and characterized their biophysical and molecular properties in accordance with the International Society of Extracellular Vesicles (ISEV) guidelines [42]. We performed a proteomic analysis and ingenuity pathway analysis (IPA) to understand the differences in their EV cargo and whether it is attributed to specific diseases and biological function pathways. Additionally, we treated HCE cells with different EV treatments to highlight if differences arise in their migratory, proliferative, and apoptotic activity. Our study shows that HCM-EVs contain protein EV cargo that may serve to enhance corneal epithelial wound healing. Understanding the differences in corneal stromal EV repertoires [43,44,45,46] will strengthen the knowledge gap associated with the molecular mechanism(s) involved in corneal wound healing.

## 2. Materials and Methods

### 2.1. Cell Culture

#### 2.1.1. Human Corneal Epithelial (HCE) Cells 

The immortalized Araki-Sasaki corneal epithelial cell line [47], HCE-TJ, was cultured and maintained in complete epithelium medium (keratinocyte-SFM (Gibco, Grand Island, NY, USA); 0.05 mg/mL bovine pituitary extract (Gibco); 5 ng/mL epithelial growth factor (Gibco); and 1× antibiotic-antimycotic (Gibco)) at 37 °C and 5% CO_2_. 

#### 2.1.2. Human Corneal Keratocytes (HCKs), Fibroblasts (HCFs), and Myofibroblast (HCMs)

Human corneas were obtained from the National Disease Research Interchange (NDRI; Philadelphia, PA, USA). All research adhered to the tenets of the Declaration of Helsinki. Cells were isolated and cultured as previously described [10,48]. Once isolated, cells were plated on 6-well plates and grown to 75% confluency in complete Eagle’s Minimum Essential Media (EMEM (American Type Culture Collection; Manassas, VA, USA) with 1× antibiotic-antimycotic (Gibco)) and either 1% (HCK media) or 10% (HCF media) fetal bovine serum (FBS, Atlanta Biologicals; Flowery Branch, GA, USA). For this study, cells exposed to 1% FBS are referred to as HCKs, denoting the maintenance, at least partially, of a normal keratocyte phenotype [49,50,51]. Cells exposed to 10% FBS are referred to as HCFs, indicating their differentiation from a normal keratocyte phenotype to a fibroblastic phenotype, as previously demonstrated [9,52]. HCMs were generated by culturing HCFs in HCF media with 2 ng/mL of TGF-β1 [9,10,11,16].

### 2.2. Extracellular Vesicle (EV) Isolation

Isolation and characterization of EVs was in accordance with the minimum information for studies of extracellular vesicles (MISEV) published in 2018 [42], as summarized by ISEV. EVs are characterized by their expression of vesicle-associated proteins (CD63, CD81, ITGAV) and diameter range of 40–150 nm. In this study, stromal cells (HCKs, HCFs, or HCMs) were cultured for 36 h in serum-free media, the conditioned media (CM) was collected, and EVs were isolated, as previously described [11]. In brief, stromal cell CM underwent serial differential centrifugation to remove cells (300× *g* for 10 min), cellular debris (3000× *g*, for 10 min), and apoptotic detritus (13,000× *g* for 30 min). The supernatant was concentrated using a Centricon^®^ Plus-70 centrifugal filter unit with a 100 kDa MW cutoff (MilliporeSigma, Burlington, MA, USA), and ultracentrifuged for 1 h and 10 min at 110,000× *g* (4 °C) using a Beckman Type 50.2 Ti Rotor (Beckman Coulter, Brea, CA, USA) in a Beckman Coulter, Optima LE-80K Ultracentrifuge. The resultant pellet was resuspended in phosphate buffered saline (PBS; Gibco), centrifuged again for 1 h and 10 min at 110,000× *g* (4 °C), and stored at −80 °C.

### 2.3. Western Blot

Protein isolation and western blot analyses were performed as previously described [7,8,12]. In brief, proteins from isolated EVs were extracted with RIPA buffer (10 mM Tris, 150 nM NaCl, 1% deoxycholic acid, 1% Triton X, 0.1% SDS, and 1 mM EDTA) plus protease inhibitors (aprotinin, PMSF, and sodium orthovanadate). Protein concentration was determined using a Pierce™ bicinchoninic acid (BCA) protein assay kit (ThermoFisher Scientific, MA, USA). Equal amounts of protein (20 μg/lane) were loaded on a 4–20% Tris-Glycine gel (Bio-Rad, Hercules, CA, USA) and electrophoresed under nonreducing conditions. Proteins were transferred to a PVDF membrane, blocked in blocking buffer (PBS, 0.05% Tween20^®^, 5% milk) for 2 h, and then probed overnight with primary antibodies: CD63 (Sc-365604; Santa Cruz, CA, USA); CD81 (Sc-7637; Santa Cruz); fibronectin (FN1) (Sc-8422; Santa Cruz); integrin αV (ITGAV) (Sc-9969; Santa Cruz); thrombospondin-1 (THBS1) (MA5-13398; ThermoFisher Scientific); and GM130 (#12480; Cell Signaling, Danvers, MA, USA). The next day, the membranes were washed and incubated for 1 h at room temperature (RT) with the following secondary antibodies: donkey anti-mouse IRDye 800CW and donkey anti-rabbit IRDye 680RD (1:2000, LI-COR Biosciences, Lincoln, NE, USA). All antibodies were diluted per manufacturer recommendation. Membranes were imaged using a fluorescence scanner (Odyssey v.3.0, LI-COR Biosciences).

### 2.4. Nanoparticle Tracking Analysis (NTA) and Zeta (ζ) Potential Measurements 

All EV samples prepared and analyzed as previously published [20,32,34]. All EV samples were diluted in PBS to a final volume of 1 mL. Ideal measurement concentrations were found by pretesting the Particle Metrix ZetaView^®^ Basic NTA PMX-120 machine (Patricle Metrix, Ammersee, Germany) at the ideal particle-per-frame value (140–200 particles/frame). The manufacturer’s default software settings for EVs/nanospheres were selected. For each measurement, three cycles were performed by scanning 11 cell positions each and capturing 30 frames per position under the following settings: focus—autofocus; camera sensitivity for all samples—75; shutter—100; scattering intensity—detected automatically; cell temperature—25 °C. After capture, the videos were analyzed by the in-built ZetaView Software 8.04.02 SP2 with specific analysis parameters: maximum area—1000; minimum area—5; minimum brightness—25; hardware—embedded laser, 40 mW at 488 nm; camera—CMOS. The number of completed tracks in NTA measurements was always greater than the proposed minimum of 1000 in order to minimize data skewing based on single large particles [53]. The zeta (ζ) potential of EVs was measured under the same settings for NTA, as described above, but the data were collected and analyzed with the ZetaView software.

### 2.5. Transmission Electron Microscopy (TEM)

EVs were fixed and imaged by transmission electron microscopy (TEM) to assess their morphology, as previously described [11,54]. Briefly, the EV pellet was resuspended in 4% *w/v* paraformaldehyde (PFA) in PBS (Gibco) and fixed for 30 min at RT. Five μl of the fixed EV solution was added to a formvar/carbon-coated grid (Electron Microscopy Sciences, Hatfield PA, CA, USA) and incubated for 20 min to allow the EVs to adhere to the grid surface. The grids were washed with drops of PBS to remove residual PFA, followed by resuspension in 1% *v/v* glutaraldehyde in PBS for 5 min. Residual glutaraldehyde was removed by gently resuspending the grid in water. The grids were transferred to a uranyl oxalate solution followed by 10 min incubation with a methylcellulose solution for contrast. Grids with adsorbed EVs were dried prior to examination by TEM (JEM-1220 TEM: JEOL USA, Peabody, MA, USA).

### 2.6. EV Labeling with PKH26

Isolated EVs were fluorescently labeled with a PKH26 Red Fluorescent Cell Linker Kit (MilliporeSigma) according to the manufacturer’s instruction [11]. The EV pellet was resuspended in diluent C, incubated with PKH26 dye in diluent C buffer at a ratio of 1:1 for 2 min at RT, and mixed with bovine serum albumin (BSA, 1% *w/v* in diluent C; Sigma Aldrich, St Louis, MO, USA) at an equal ratio per volume. The PKH26–EV solution was subjected to ultracentrifugation using a Beckman Type 50.2 Ti Rotor (Beckman Coulter) in an Optima LE-80K Ultracentrifuge (Beckman Coulter) at 110,000× *g* for 1 h and 10 min at 4 °C. The supernatant was removed and the PKH26-labeled EVs were washed, resuspended in diluent C, and ultracentrifuged (110,000× *g* for 1 h and 10 min at 4 °C). The wash/ultracentrifugation steps were repeated a total of three times. PKH26-labeled EVs were filter-sterilized (0.22 μm pore) prior to use in cell culture. For the control sample, particle-free PBS (Gibco) was used instead of EVs and stained according to the procedures described above.

### 2.7. Tracking PKH26-Labeled EVs in HCE Cells 

HCE cells were cultured in a 12-well plate at a density of 0.1 × 10^6^ cells per well and incubated at 37 °C and 5% CO_2_ for 24 h. Following attachment, HCE cells were serum starved in keratinocyte-SFM devoid of supplements for 24 h. Following serum starvation, cells were washed with PBS (Gibco), stained with 1 μg/mL Hoechst 33342 (ThermoFisher Scientific) for 1 min at RT, washed again with PBS, and supplemented with keratinocyte-SFM media containing PKH26-labeled EVs. Live cell imaging with the Leica DMi8 microscope (Leica Microsystems, Wetzlar, Germany) began 1 h post-administration of PKH26-labeled EVs and continued for 24 h, with images and videos obtained every 15 min. Endpoint images and videos were analyzed by ImageJ (Version 1.53n7; National Institutes of Health (NIH), Bethesda, MD, USA) analysis. Image sequences (Hoechst-positive cells) were imported, converted to greyscale, and analyzed with the Trackmate plugin. All Hoechst-positive cells were measured (25 pixel constant diameter), and mean intensity values were obtained. Mean velocity and displacement of the cell per pixel were acquired following the removal of any outliers.

### 2.8. In Vitro HCE Cell Scratch Assay 

HCE cells (20,000 per well) were plated in 48-well plates and cultured to confluency in complete epithelial medium. Once confluent after 48 h, HCE cells were serum starved in keratinocyte-SFM devoid of supplements for 24 h. A linear scratch wound was performed using a 200 μL pipette tip across the center of each well, and images of the scratch area (0, 12, and 24 h after wounding) were captured using an inverted microscope with a 10× objective lens (EVOS XL Core Imaging System: Life Technologies, Bothell, WA, USA). The images were analyzed and the remaining wound area was measured using ImageJ software. All values were normalized to wounds at 0 h. 

### 2.9. Tandem Mass Tag (TMT) Mass Spectrometry

#### 2.9.1. Experimental Design and Statistical Rationale 

All TMT Mass Spectrometry experiments were performed by BGI Genomics (BGI Genomics, San Jose, CA, USA). All EVs were lysed, protein concentrations were measured, and enzymatic digestion was applied, akin to western blotting. All 3 samples were labeled with a Tandem Mass Tag™ (TMT™) 10-plex for quantitative proteomics experiment. After the labeling step, a small fraction of each sample was pooled together to examine the labeling efficiency. 

#### 2.9.2. Sample Preparation and Proteolytic Digestion

Sample preparation and proteolytic digestion were performed as per the previously published protocols by BGI Proteomics [55,56,57]. For stromal EV sample lysis and preparation, RapiGest (Waters, Milford, MA, USA) was added to each EV sample to a final concentration of 1% RapiGest (mass/volume). Then, the samples were sonicated for 10 s at 20% amplitude using a Q500 Sonicator (QSonica, Newtown, CT, USA), heated at 90 °C for 5 min, centrifuged, and measured for protein concentration with a BCA assay (ThermoFisher Scientific). For proteolytic digestion, equal amounts of protein (15 µg) for each stromal EV sample were normalized to the same volume of 400 μL with 100 mM (pH 8.5) HEPES buffer, 40 μL of 10 mM dithiothreitol (DTT) was added to each sample and incubated at 60 °C for 15 min in an Eppendorf Thermomixer^®^ C (Eppendorf, Hamburg, Germany), and then 40 μL of 20 mM iodoacetamide (IAM) was added and incubated at RT for 20 min in the dark. Samples were quenched with 10 mM DTT to eliminate excess IAM. To bring the RapiGest concentration below 0.5% for enzymatic digestion, 450 μL H_2_O and 30 μL 100 mM HEPES buffer (pH 8.5) were added to each sample, and then 15 μL of 0.1 μg/μL trypsin/LysC (ThermoFisher Scientific) was added and the samples were shaken and incubated for 12 h at 37 °C in an Eppendorf Thermomixer^®^ C. Five μL of trypsin/LysC was added, and the samples were shaken and incubated for 16 h. To quench the trypsin reaction, 100 μL of 10% trifluoroacetic acid (TFA) was added to each sample [58]. Each sample was purified with C18 stage tips (PhyNexus Inc, San Jose, CA, USA) and elutes from each sample were dried down in the Speedvac SPD120 concentrator (ThermoFisher Scientific) for TMT labeling.

#### 2.9.3. TMT Labeling and Label Check

Each dried sample was resuspended in 40 μL of 100 mM HEPES (pH 8.5), gently vortexed, and centrifuged. Three new specific TMT labels were resuspended in 80 μL acetonitrile (ACN; Optima™ LC/MS Grade; ThermoFisher Scientific), and 20 μL of TMT reagent was added to each EV sample. All samples were incubated at RT for 1 h [59]. To confirm the TMT labeling [21], 1 μL of each TMT-labeled sample was removed and transferred into 80 μL reconstitution buffer (1% formic acid), mixed, and analyzed by liquid chromatography–mass spectrometry (LC/MS), which indicated labeling efficiency to be ~99%. To quench the TMT reaction, 15 μL of 5% hydroxylamine solution was added to each TMT-labeled sample. TMT-labeled samples were then mixed and dried down in the Thermo Speedvac SPD120 (ThermoFisher Scientific).

#### 2.9.4. High-pH Reverse-Phase High-Performance Liquid Chromatography (HPLC)

Dried TMT-labeled samples were reconstituted with 80 μL deionized water and fractionated by high-pH reverse-phase high-performance liquid chromatography (HPLC; ThermoFisher Scientific). A total of 96 fractions were collected over 75 min. These initial fractions were combined to form 8 final fractions; therefore, 12 fractions were combined into 1 fraction. Combined fractions were dried down and desalted using C18 stage tips (PhyNexus Inc).

#### 2.9.5. Liquid Chromatography–Tandem Mass Spectrometry (nanoLC–MS/MS)

NanoLC–MS/MS was performed as per the previously published protocols by BGI Proteomics [55,56,57]. All fractionated samples were analyzed by Ultimate™ 3000 nanoflow HPLC (ThermoFisher Scientific) followed by Orbitrap Eclipse Tribrid Mass Spectrometry (ThermoFisher Scientific). The Nanospray Flex™ Ion Source (ThermoFisher Scientific) was equipped with a PRSO-V2 Column Oven (Sonation, Biberach, Germany) to heat up the PicoFrit^®^ nanocolumn (100µm × 250 mm × 15µm tip; New Objective, Littleton, MA, USA) for peptide separation. The nanoLC method is water/ACN-based, with a 0.35 μL/min flowrate (150 min). For each TMT fraction, all TMT-labeled peptides were first engaged on a trap column (ThermoFisher Scientific) and then delivered to the separation nanocolumn by the mobile phase. A TMT-specific MS2-based MS method on Orbitrap Eclipse was used to sequence TMT peptides that were eluted from the nanocolumn. For the full MS, 120,000 resolution was used with 3E6 AGC target, and the scan range was 300–1500 m/z. For the dd-MS2(MS/MS), 60,000 resolution was used with 1E5 AGC target. Isolation window was 0.7 Da, with a fixed first mass of 110.0 Da. Normalized collision energy was set to 32, with a 15-cycle loop.

#### 2.9.6. Quantitative Proteomic Analysis

Collected LC–MS data was analyzed by Proteome Discoverer 2.4 (ThermoFisher Scientific). As all peptides were labeled with TMT tags, TMT quantitative proteomic searches were performed utilizing SEQUEST HT node with mass tolerance of 20 ppm MS1 and 0.05 Da for MS2. Homo Sapiens database (UP000005640) from Swiss-Prot was used, and percolator node was used for peptide false discovering rate (FDR) filtering (stricted—001; relaxed—0.05). TMT-labeled peptide abundance was normalized by total peptide abundance.

#### 2.9.7. Ingenuity Pathway Analysis (IPA) of EVs

Using IPA software (Qiagen, Germantown, MD, USA), proteins involved in disease function pathways related to cellular movement, growth, proliferation, and development were explored. Proteins that exhibited a false-discovery-rate adjusted *p*-value <0.05 and a log-fold change >0.5 or <−0.5 between HCK-EVs vs. HCM-EVs and HCF-EVs vs. HCM-EVs were uploaded onto IPA for primary analysis. Canonical, disease, and function pathways for the pairwise comparisons for each group were then combined into a final analysis using the IPA comparison module. Only pathways showing a Z score >2 or <−2 among the final combined pairwise comparisons were included, among which positive and negative Z scores represented activated and inhibited pathways, respectively. Protein lists for relevant pathways were then downloaded from IPA for further analysis and visualization into heat map configuration.

### 2.10. Statistics

Data were reported as mean ± SEM unless stated otherwise. Differences between groups were compared by ANOVA followed by Tukey’s multiple comparison test using GraphPad Prism (Version 8.4.2; GraphPad, CA, US). *p* values < 0.05 were considered significant: * *p* < 0.05, ** *p* < 0.01, *** *p* < 0.001, **** *p* < 0.0001.

## 3. Results

### 3.1. Characterization of HCK-, HCF-, and HCM-EVs

**Figure 1:**
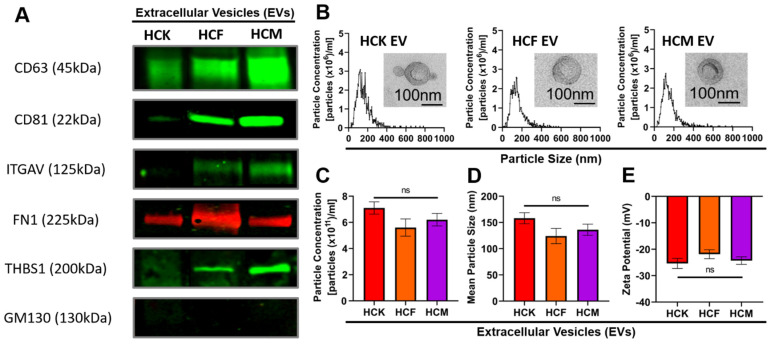
Isolation and characterization of human corneal keratocyte (HCK)-, fibroblast (HCF)-, and myofibroblast (HCM)-derived extracellular vesicles (EVs). (**A**) EV pellets (20 μg protein/lane) were analyzed by western blot to probe for vesicle-associated markers. Representative images are shown for vesicle-associated proteins CD63, CD81, and ITGAV; a negative control (GM130); and proteins associated with corneal wound healing, FN1 and THBS1. (**B**) EV pellets were analyzed by nanoparticle tracking analysis (NTA), and a size distribution histogram for each EV sample is shown. ((**B**), inset) Transmission electron microscopy images demonstrating EV morphology (high magnification, 49000x; scale bar = 100 nm). (**C**) The average particle concentrations (particles x1011/mL); (**D**) mean particle size (nm); and (**E**) zeta (ζ) potential of EV pellets were measured using NTA with Zetaview™. Data are shown as mean + SEM; *n* = 3 independent EV preparations; ns = nonsignificant. One-way ANOVA with Tukey’s post-test.

To investigate whether EVs were isolated successfully from HCKs, HCFs, and HCMs, we used an established EV isolation protocol [11,16]. We adhered to the ISEV guidelines for EV characterization by utilizing multiple approaches [42]; EVs in this study were normalized to the protein concentration. We performed western blotting by probing for vesicle-associated markers: CD63, CD81, and ITGAV (Figure 1A). HCF- and HCM-EVs showed high expression levels of CD63 and CD81, but this was decreased in HCK-EVs. Additionally, HCF-EVs and HCM-EVs showed elevated ITGAV expression compared to HCK-EVs.

We also probed for the cornea wound-healing proteins FN1 and THBS1. HCM-EVs showed abundant THBS1 expression, with this decreasing in HCF-EVs and HCK-EVs, respectively. Interestingly, HCF-EVs showed elevated FN1 expression compared to HCK-EVs and HCM-EVs. All EV preparations showed negative GM130 expression, indicative of no contaminants. To validate the biophysical properties of EVs, we performed NTA measurements to show the size distribution of the particles (Figure 1B), and the EV morphology by TEM (Figure 1B, inset) was similar, irrespective of cell type. We found no differences in the particle concentration measurements of EVs ranging from 5.6 × 10^11^ to 7.1 × 10^11^ particles/ml (Figure 1C); a mean particle size ranging from 124 to 158 nm (Figure 1D); and a net negative surface charge ranging from −21.87 mV to −25.38 mV, which is indicative of colloidal EV stability (Figure 1E). Collectively, the data suggested differences in protein expression, while we saw negligible effects in their biophysical properties.

### 3.2. Proteomic Analysis of HCM-EV Protein Cargo

**Figure 2:**
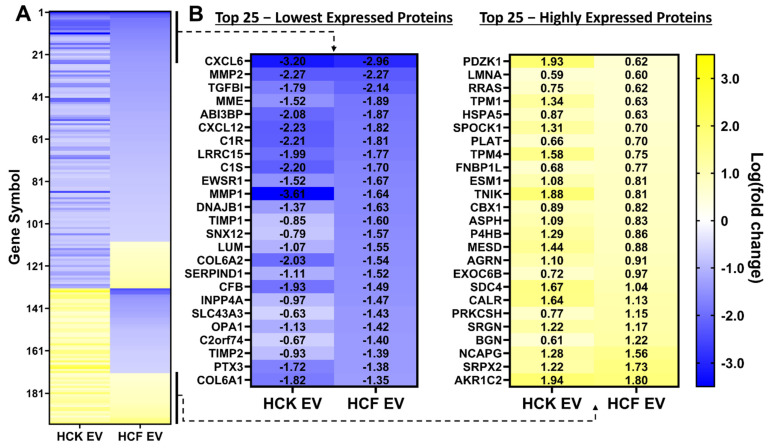
Proteomic analysis of corneal keratocyte, fibroblast, and myofibroblast extracellular vesicles. (**A**) The heatmap of differentially expressed proteins from human corneal keratocyte (HCF) and fibroblast (HCF) relative to myofibroblast (HCM) extracellular vesicles. A multigroup comparison was performed and showed that 195 proteins were differentially expressed in our dataset; proteins selected show log-fold change values of target proteins (blue (−3.5) to yellow (+3.5) through white), ranked by adjusted *p* < 0.05. (**B**) The top 25 proteins are selected from this heatmap with decreased (blue (−3.5)) and increased (yellow (+3.5)) log-fold change in HCK-EVs and HCF-EVs relative to HCM-EVs. Data shown as *n* = 3 independent EV preparations.

To compare EV protein cargo from HCK-, HCF- and HCM-EVs (pooled from three independent preparations), samples were digested in-solution and analyzed by nanoLC–MS/MS. In total, peptides from 2482 unique proteins were identified from all EV samples, and further details are specified in Table S1. Peptides with a log fold change <−0.5 or >0.5 between protein profiles from HCM-EVs vs. HCK-EVs or HCM-EVs vs. HCF-EVs were confirmed. A multigroup comparison was performed and showed 195 proteins were found to have significant log-fold changes (as described) based on a false-discovery-rate (FDR) adjusted *p*-value of <0.05 (Figure 2A). Whilst the magnitude of change was dissimilar for many proteins, the presented heatmap (Figure 2B) narrows that number and lists the top 25 least expressed proteins with a <−0.5 log-fold decrease in HCK-EVs or HCF-EVs when compared to HCM-EVs, such as CXCL6, CXCL12, LRRC15, MMP1, MMP2, and TGFBI. Additionally, we extended our observations to highlight the top 25 most highly expressed proteins with a >0.5 log-fold increase in HCM-EVs compared to HCK-EVs or HCF-EVs, such as TNIK, LMNA, CALR, and PLAT. The data show similarities across the different EV samples but demonstrate where the EV cargo is diverse in protein expression.

### 3.3. IPA of HCM-EV Proteins

**Figure 3:**
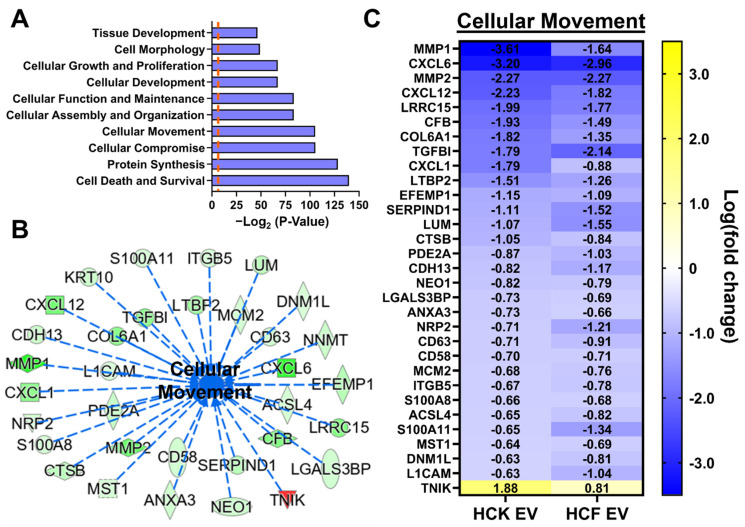
Ingenuity pathway analysis of proteins from corneal myofibroblast extracellular vesicles. (**A**) The top 10 disease and biological functions identified from the ingenuity pathway analysis (IPA) using the dataset of 195 proteins. The *p*-value for each biological function is indicated by the bar and is expressed as -log_2_ (*p*-value). The orange dashed line indicates the threshold of significance (*p* < 0.05). (**B**) The top-scoring proteins identified by IPA for cellular movement; green indicates decreased measurement, red indicates increased measurement, blue dashed arrow leads to inhibition. (**C**) The heatmap shows 31 proteins with decreased (blue (−3.5)) and increased (yellow (+3.5)) log-fold change in HCK-EVs and HCF-EVs relative to HCM-EVs. Data shown as *n* = 3 independent EV preparations.

To gain a functional insight into the proteomic cargo of HCM-EVs, we followed the initial selection step to show log-fold changes <−0.5 or >0.5 that showed statistically significant differences in proteins from HCM-EVs compared to HCK-EVs or HCF-EVs. From the shortlisted proteins (Figure 2), we performed ingenuity pathway analysis (IPA) for the top disease and biological functions enriched in HCM-EVs. The results show the top 10 biological functions via IPA associated with these differentially expressed proteins in HCM-EVs, which include cell death and survival (-log (*p*-value = 139.757)), cellular movement (-log (*p*-value = 105.439)), cellular growth and proliferation (-log (*p*-value = 67.141)), and tissue development (-log (*p*-value = 46.429)), as some functional examples (Figure 3A). Of interest, increased cellular movement in HCM-EVs compared to HCF-EVs or HCK-EVs via IPA analysis revealed changes in protein expression, as listed in Figure 3B. Within the HCM-EV heatmap dataset, we identified CXCL1, CXCL6, CXCL12, MMP1, MMP2, L1CAM, and LRCC15 as proteins influencing cellular movement, cell proliferation, and tissue development compared to HCF-EVs or HCK-EVs (Figure 3C).

### 3.4. HCM-EVs Promote HCE Cell Migration

**Figure 4:**
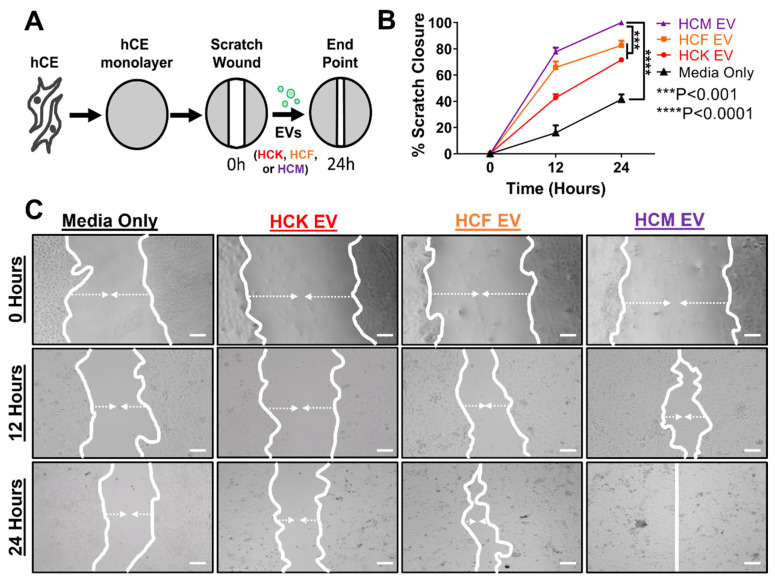
Corneal myofibroblast extracellular vesicles accelerate corneal epithelial cell motility. (**A**) Schematic of human corneal epithelial (HCE) cells treated by corneal stromal-derived extracellular vesicles (EVs) in an in vitro scratch assay. A monolayer of growth-arrested HCE cells were freshly scratched using a 200 μL pipette tip. After 24 h, HCE cells were treated with EVs derived from human corneal keratocytes (HCKs), fibroblasts (HCFs), and myofibroblasts (HCMs) (normalized for protein concentration). The closure of the scratch was monitored microscopically up to 24 h thereafter. (**B**) Measurements of the scratch width were taken throughout the time course and are plotted as the proportion of scratch width relative to that at scratch initiation (0 h) at each time point. (**C**) Representative images of wells at scratch initiation, at 12 h, and at 24 h are shown, and the margin of scratch is emphasized by the white lines. White arrows depict movement of scratch. Scale bar: 100 μm. Data are shown as mean + SEM; *n* = 4 per group. Two-way ANOVA with Bonferroni post-test.

It has been reported that upon corneal injury, re-epithelialization is one key aspect of the route towards corneal epithelial wound healing. After investigating the differences in protein expression in HCK-, HCF- and HCM-EVs, we next assessed the ability of these EVs to promote corneal epithelial cell migration. A monolayer of confluent HCE cells was established, and once confluent, they were growth-arrested to remove promotility factors, followed by the formation of a vertical scratch. Using a protein-dependent EV normalization approach, HCK-, HCF-, and HCM-EVs were added, and we tracked the closure of a single scratch over 24 h (Figure 4A). Compared with the HCM-EVs, which achieved complete closure over 24 h, the HCF-EV and HCK-EV treatments achieved incomplete closure (82.64% ± 3.67% and 71.61% ± 0.96%, *p* < 0.001, respectively) (Figure 4B). Nevertheless, the closure rate remained significantly slower when using media (without growth factors) (41.50% ± 3.81%, *p* < 0.0001). Representative brightfield images at scratch initiation, at 12 h, and at 24 h with different treatments are shown in Figure 4C. Additionally, this suggests that HCM-EVs accelerate HCE cell migration compared to HCF-EVs and HCK-EVs.

### 3.5. HCM-EVs Increase HCE Cell Proliferation

**Figure 5:**
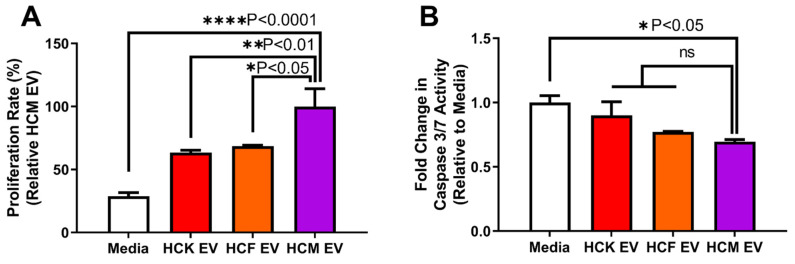
Corneal myofibroblast extracellular vesicles increase corneal epithelial cell proliferation. A monolayer of growth-arrested human corneal epithelial (HCE) cells were scratched using a 200 μL pipette tip after 24 h following EVs derived from human corneal keratocytes (HCKs), fibroblasts (HCFs), and myofibroblasts (HCMs) (normalized for protein concentration). Cell-conditioned media were removed and replenished with supplement-free keratinocyte-SFM media. Treated HCE cells were assessed with (**A**) WST-8 reagent (absorbance values = 450 nm) to assess cell proliferation and (**B**) Caspase-Glo^®^ 3/7 reagent (luminescence) to assess apoptotic activity. Data are shown as mean + SEM; *n* = 3 per group. One-way ANOVA with Tukey’s post-test.

Considering the differences in the migratory capacity of HCE cells from different EV treatments, we next determined the proliferative capacity of the treated HCE cells by WST-8 absorbance. HCM-EV treatment showed a significant difference compared to media alone (no supplements) following 24 h EV treatment (100.00% ± 8.11% vs 28.82% ± 1.66%, *p* < 0.0001, (Figure 5A)). Similarly, we observed that HCK-EV (63.40% ± 1.12%, *p* < 0.01) and HCF-EV (68.57% ± 0.52%, *p* < 0.05) proliferation was significantly reduced compared to the HCM-EV treatment after 24 h. To explore the apoptotic activity, we used a Caspase-Glo^®^ 3/7 reagent to measure the luminescence from the treated HCE cells with different EVs. The HCM-EV treatment significantly reduced the levels of apoptotic activity when compared to the media treatment with no supplements (0.69 ± 001 vs. 1.00 ± 0.04, *p* < 0.05 (Figure 5B)). Interestingly, we saw minimal apoptotic differences in the HCK-EV (0.90 ± 0.07, *p* > 0.05) and HCF-EV (0.78 ± 0.01, *p* > 0.05) treatments compared with HCM-EV. These data suggest that HCM-EVs promote HCE cell proliferation with a minimal impact upon apoptosis.

### 3.6. PKH26-Labeled HCM-EVs Promote Cell Velocity

**Figure 6:**
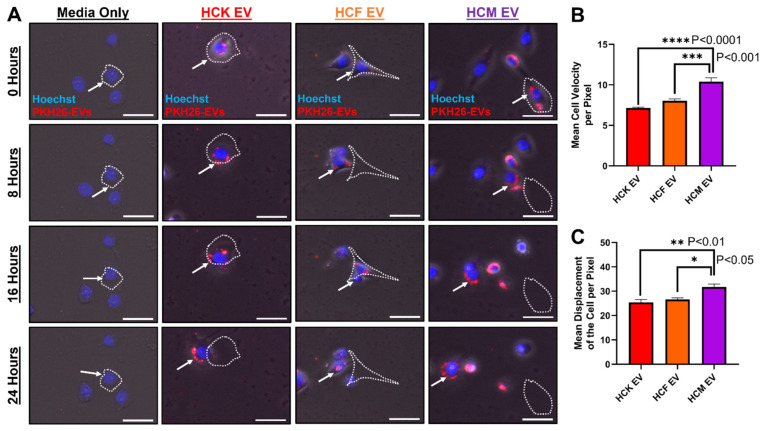
PKH26-labeled corneal myofibroblast extracellular vesicles increase corneal epithelial cell velocity. (**A**) Human corneal epithelial (HCE) cells were seeded into a 12-well plate and growth-arrested for 24 h. Attached HCE cells were stained with Hoechst 33342. EVs derived from human corneal keratocytes (HCKs), fibroblasts (HCFs), and myofibroblasts (HCMs) were labeled with the lipophilic dye PKH26. PKH26-labeled EVs (red) were added to Hoechst-33342-stained HCE cells (blue), and images were obtained at 0, 8, 16, and 24 h. White dashed outline indicates initial cell size and location at 0 h. The white arrow indicates PKH26-labeled EVs. Scale bar: 50 μm. (**B**) Images and videos were collected at the endpoint and analyzed by ImageJ. Image sequences (Hoechst-positive HCE cells) were imported and converted to greyscale images. Analysis occurred using the Trackmate plugin to acquire mean cell velocity and (**C**) mean displacement of the cell per pixel. Data are shown as mean + SEM; *n* = 3 per group. One-way ANOVA with Tukey’s post-test.

Considering the differences we observed with HCM-EVs in promoting HCE migration and proliferation, we further investigated whether HCM-EVs can promote HCE velocity. We stained growth-arrested HCE cells with Hoechst 33342 and treated these cells with PKH26-labeled HCK-, HCF- and HCM-EVs. We tracked the movement of Hoechst-positive HCE cells and PKH26-labeled EVs hourly for 24 h, and representative images are shown in Figure 6A. We showed that treatment with PKH26-labeled HCM-EVs (10.40 ± 0.47) significantly increased the HCE cell velocity per pixel when compared to HCK-EVs (7.14 ± 0.12, *p* < 0.0001) or HCF-EVs (8.03 ± 0.24, *p* < 0.001) (Figure 6B). The media-only treatment showed negative PKH26-positive particles, indicative of no free-floating PKH26 particles. Furthermore, the HCM-EV (31.72 ± 1.28) treatment significantly increased the mean displacement of HCE cells per pixel when compared to HCK-EV (25.40 ± 1.18, *p* < 0.01) and HCF-EV (26.62 ± 0.66, *p* < 0.05) (Figure 6C).

## 4. Discussion

Corneal wound repair is a complex process involving interactions between the wound-healing epithelium, a temporary “provisional matrix”, and the stromal cells [3,13,14]. During corneal injury, the regulated changes of stromal cell phenotypes are mediated by a complex web of biophysical and chemical cues [48,60,61]. Specifically, the onset of HCMs by TGF-β1 or -β3 plays a vital role in wound healing in the contraction and closure of incisional corneal wounds and surface re-epithelialization [62]. Studies have acknowledged that the secretome of stromal cells contributes to reinforcing the injured site in either a regenerative manner or in a pathological fibrotic manner [38,63,64]. However, there persists a knowledge gap regarding the stromal paracrine-EV mechanism(s) and how the protein EV cargo is of most relevance in corneal epithelial wound healing, as this remains unexplored to date.

In our present study, we isolated HCK-, HCF-, and HCM-EVs and characterized their biophysical and molecular properties. We showed the differences in their molecular properties by detecting the CD63 in all the corneal stromal EVs, but found elevated CD81 and ITGAV expression in the HCF-/HCM-EVs. Additionally, we showed the differences in THBS1 and FN1 expression in all the preparations, but all the EVs showed negative GM130 levels, indicative of no contaminants [65,66]. Although some molecular differences were present, we examined the considerable overlap in their biophysical properties when assessing the EV morphology, size distribution measurements, particle concentration, and zeta potential. These data suggest that we isolated and characterized EVs in accordance with the ISEV requirements.

Following the isolation of HCK-, HCF-, and HCM-EVs, there are many normalization strategies varying between cell number, lipid concentration, or particle counts, but we kept a constant protein concentration, akin to previous corneal-related studies [11,16,41], as this eliminates any bias towards overdosing EV towards a different metric. Hence, the corneal stromal EVs used for the proteomic analysis and functional experiences were normalized by protein concentration and should reflect the differences in EV phenotype.

Our proteomics study showed that HCM-EVs had a distinct EV protein cargo profile compared to HCK- and HCF-EVs, with 195 proteins being significantly different. We highlight the elevated expression of HCM-EV proteins such as CD63, CDH13, CTSB, CXCL1, CXCL6, CXCL12, LRRC15, MMP1, and MMP2 from our proteomic analysis. Collectively, from our IPA analysis, these proteins are proposed to affect cellular movement, but also pathways related to cell proliferation, epithelial tissue development, and growth. Studies suggests that CXCL1 and CXCL12 are components shown to accelerate intestinal, pulmonary, or ovarian epithelial cell migration [67,68,69]. Similarly, the overexpression of CXCL1, CXCL6, or CXCL12 has been shown to increase epithelial cell migration, proliferation, and growth, albeit in many cancer models [68,70,71,72,73,74]. Of relevance to the eye, CXCL12 expression plays a role in the development and formation of ocular tissue in chick and mouse models [75]. Additionally, MMP1 and MMP2 can enhance cellular growth and migration in cancer [76,77,78,79,80] and wound-healing models [81,82,83]. Of interest to the cornea, MMP1 and MMP2 have been shown to increase HCE cell migration [60,84] and can aid corneal wound healing [85]. This reveals an important difference between HCK-, HCF-, and HCM-EVs in terms of their protein cargo, as it highlights that increased CXCL1, CXCL6, CXCL12, MMP1, and MMP2 expression is likely to influence epithelial cell migration, proliferation, and growth functions.

We used a well-established in vitro corneal scratch assay [86,87,88,89], in which HCE movement is dependent upon EV stimulus and can generate a strong directional migratory response [90,91]. As such, we provided evidence that the HCM-EV treatment accelerated HCE cell migration and motility compared to the HCK- and HCF-EV treatments. This is further supported by the increased HCE proliferation resulting from the HCM-EV treatment when compared to the other EV treatments. Similar to our data are observations that HCM secretome can promote wound-healing mechanisms in different models [63,92,93], thus agreeing with the premise that the HCM EV-secretome in part plays a role in promoting corneal epithelial wound healing. Despite the evidence, the HCM-EV cargo remains elusive and investigation into its interactions with other corneal cells warrants the distinction of the critical elements that can dictate the potency of corneal epithelial wound healing.

There were several limitations in this study, which in future studies will be explored. Some experiments were performed with the HCE cell line; we acknowledge that future studies and investigations do require the translation of our findings into using primary cell lines, as they are more physiologically relevant compared to immortalized cell lines, despite the ease of cell culture and proliferation. We did not evaluate in depth other EV cargo proteins that could be pivotal for biological functions, such as tissue development or cellular compromise. The data at hand show HCM-EV cargo proteins that could be pivotal for accelerating corneal epithelial wound healing, and although this can only be deemed as speculative, this does warrant further investigation. Therefore, to provide a greater in-depth understanding, within our future studies we should employ different types of short interfering/short hairpin RNA (si/shRNA)-silencing approaches to address the specific key EV cargo that can accelerate corneal epithelial migration and wound healing. Furthermore, in this study, we focused on the interaction of HCM-EV cargo with the corneal epithelia, yet we cannot dismiss the possibility that the cargo can exert different effects on the cornea, such as the stroma, immune system, and lacrimal glands, which could potentially exacerbate the fibrotic or inflammatory responses.

In addition, we reported that the HCM-EV secretome can accelerate epithelial cell migration, motility, and proliferation, yet other studies have reported that myofibroblast soluble secretome can exert similar migratory capacity and tissue microenvironmental changes [61,63,64,94,95]; however, there are minimal studies on HCM-EVs. We also note that HCM-soluble secretome could modulate HCE cell migration, and this prospect cannot be excluded, though any conjecture on the topic can only be deemed as speculative. Future studies will explore and compare protein levels between the soluble factors and EVs, as these components remain to be fully understood.

The current EV study provides evidence that HCM-EVs compared to HCK-/HCF-EVs contain distinct cargo proteins that can promote HCE cell migration, proliferation, and motility. The proteins identified here could be elements contributing to corneal epithelial wound healing by the expression of CXCL1, CXCL6, CXCL12, MMP1, or MMP2 by EVs, and hence may provide proteins of interest that could be harnessed towards accelerating cornea wound closure. Though deciphering in detail the molecular components and mechanism(s) of action will be important for future studies, we demonstrated that the EV cargo is distinct but could provide beneficial findings to accelerate corneal epithelial wound healing.

## Data Availability

Not applicable.

## Supplementary Materials

The following supporting information can be downloaded at: https://www.mdpi.com/article/10.3390/ijms23063136/s1.
